# Clinical practice with antidementia and antipsychotic drugs: Audit from a geriatric clinic in India

**DOI:** 10.4103/0019-5545.58292

**Published:** 2009

**Authors:** Krishna Prasad, Himanshu Gupta, Srikala Bharath, Om Prakash, P. T. Sivakumar, C. Naveen Kumar, Mathew Varghese

**Affiliations:** Department of Psychiatry, National Institute of Mental Health and Neurosciences, Bangalore - 560 029, India; 1 Department of Psychiatry, Institute of Human Behaviour & Allied Sciences, Delhi - 110 095, India

**Keywords:** Antidementia drugs, antipsychotics, dementia, prescription pattern

## Abstract

**Background:**

Dementia is one of the most disabling disorders afflicting the elderly, with a staggering emotional and economic impact. Antidementia agents have been used for delaying cognitive decline. Antipsychotics are commonly prescribed for behavioral symptoms associated with dementia.

**Objectives:**

To explore the use of anti-dementing agents and antipsychotics used in patients with a diagnosis of dementia

**Materials and Methods:**

A retrospective chart review method; geriatric clinic of tertiary care setting.

**Results:**

The study sample included 51 consecutive patients with a diagnosis of dementia. The commonest subtype of dementia that was diagnosed was Alzheimer's disease (45%), followed by Frontotemporal dementia (25%).The commonest antidementia drug that was used was donepezil, which alone was prescribed in 27 patients (52%). The commonest antipsychotic used was quetiapine, which was used in 24 patients (47%).

**Conclusions:**

The study found donepezil to be the most commonly prescribed antidementia drug and quetiapine to be the most commonly used antipsychotic in a tertiary care geriatric clinic, in a developing country. There is a need to study the cost-effectiveness of antidementia and antipsychotic drugs in patients with dementia, in developing countries.

## INTRODUCTION

Dementia is a silent epidemic afflicting the elderly with a staggering emotional and economic impact, throughout the world, including India. The Delphi consensus study[1] estimated its prevalence to be 1.9% in people above 60 years of age in India and South Asia, with an annual incidence of 4.3/1000. A majority of dementia patients live in third-world countries, representing an under-recognized public health burden. Economic constraints further add to problems in the availability of institutionalized elderly care and a state-sponsored health care system. Evidence-based practice for the management of dementia is also hampered by the lack of clear-cut expert consensus on the efficacy of antidementia drugs.[2]

The treatment of dementia varies through the course of the illness, because symptoms evolve over time. Cholinesterase inhibitors that include donepezil, rivastigmine, and galantamine have been recommended for use in mild-to-moderate Alzheimer's dementia. Memantine, a noncompetitive N-methyl d-aspartate (NMDA) antagonist, is the only drug that has been recommended for use in severe dementia, with evidence supporting its use in moderate dementia.[3] Appropriate utilization of antidementia therapy and care management is vitally important for achieving quality of life and care for dementia patients and their caregivers, and for managing the excess costs of Alzheimer's disease.[4]

Behavioral and psychological symptoms of dementia (BPSD) are an integral part of the dementia syndrome. Psychosis, agitation, and other behavioral symptoms are reported in patients with dementia causing significant caregiver distress and leading to early institutionalization.[5] The general key elements in the management of BPSD are clarification of target symptoms, ruling out delirium and co-morbid major psychiatric diagnoses, and creatively addressing possible social, environmental, or behavioral remedies. The atypical antipsychotics (Olanzapine, Quetiapine, Risperidone, and Clozapine) have become first-line pharmacological treatments for behavioral symptoms in patients with dementia, because of the perception that they are safer and more effective when compared to the typical antipsychotic medications.[6] However, the increased risk of mortality reported with the use of atypical antipsychotics has resulted in considerable skepticism over their use for behavioral and psychotic symptoms.

The prescription pattern of antidementia drugs and antipsychotics in dementia patients has not been studied in developing countries where cost may be the most important factor in determining their choice. We therefore studied retrospectively, patients with cognitive complaints presenting to the tertiary care geriatric clinic of our hospital with an aim to investigate the use of antidementia and antipsychotics drugs prescribed to dementia patients.

## MATERIALS AND METHODS

### Study design and sample

The study reported in this article was conducted at the Geriatric Clinic Outpatient Department of a tertiary center (NIMHANS, Bangalore, India). This study is a clinical audit based on a retrospective chart review of 51 individuals with an ICD-10 (International Classification of Diseases and related health problems, tenth revision)[7] diagnosis of dementia and who received outpatient treatment from this center between January and December 2007. This was an open, observational study, with an incidental sample (since this was an audit and not a trial).

In all patients, the diagnosis was made by a detailed, face-to-face, semi-structured clinical interview by resident doctors in psychiatry utilizing the ICD - 10 criteria.[7] After a detailed workup, the diagnoses were confirmed by independent review and an interview with the patient by one of the four consultant psychiatrists (MV, SB, OP, and PTS), as the standard practice of care in our clinic.[8]

The bulk of this study focused on the detailed reading of individual case files of patients. Initially, lists of individuals with ICD-10 diagnosis of dementia were identified from the outpatient services of the geriatric clinic. After identification, these case notes were reviewed, and relevant information was incorporated in the study, mainly focusing on the antidementia and antipsychotic drugs prescribed. The data were suitably coded and extracted on a custom Microsoft excel sheet and analyzed. As this was a chart review of patients already in treatment with voluntary consent, individual informed consent was not obtained.

## RESULTS

### Sample characteristics

Fifty-one consecutive patients with dementia, who registered at the Geriatric Clinic Outpatient Department of a tertiary center, met the study criteria for the study sample. The mean age of the sample was 65.72 years. The sample included 23 males (45.1%) and 28 females (54.9%). A majority of the patients belonged to the middle-socioeconomic status (57%).

The commonest subtype of dementia that was diagnosed was Alzheimer's disease (45%), followed by fronto-temporal dementia (25%), followed by others,[Table 1]. The HMSE (Hindi Mental State Examination)[9] scores of more than two-thirds of the sample were below 19 and a quarter of the sample had scores below 10 [Table 2].

**Table 1:** Subtypes of diagnosed dementia cases

Diagnosis	Number of cases (%)
Alzheimer's disease	23 (45)
Vascular dementia	6 (12)
Fronto-temporal dementia	13 (25)
Mixed dementia	3 (6)
Diffuse lewy body disease	2 (4)
Other dementias	4 (8)

**Table 2:** Dementia cases and their HMSE scores

HMSE scores	Number of cases (%)
Less than 10	13 (25)
10 to 18	20 (39)
19 to 23	3 (6)
24 to 31	6 (12)
Not available	9 (18)

HMSE = Hindi Mental State Examination)[9]

### Antidementia drugs used

**Figure 1:**
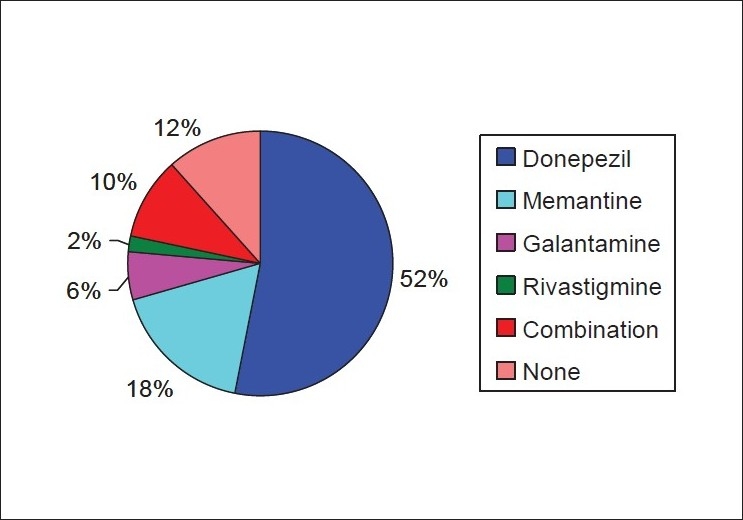
Pie diagram depicting the antidementia drugs used in patients with dementia

The commonest antidementia drug that was used was donepezil, which alone was prescribed in 27 patients (52%). It was prescribed in a dose range of 5 to 10 mg/day. Memantine alone was prescribed in nine patients (18%). A combination of choline-esterase inhibitor and memantine was used in five patients (10%). Galantamine alone was used in three patients only, whereas, Rivastigmine alone was used in a single patient. Six patients (12%) were not prescribed any antidementia drugs. The findings are depicted in Figure 1.

### Antipsychotics used

**Figure 2:**
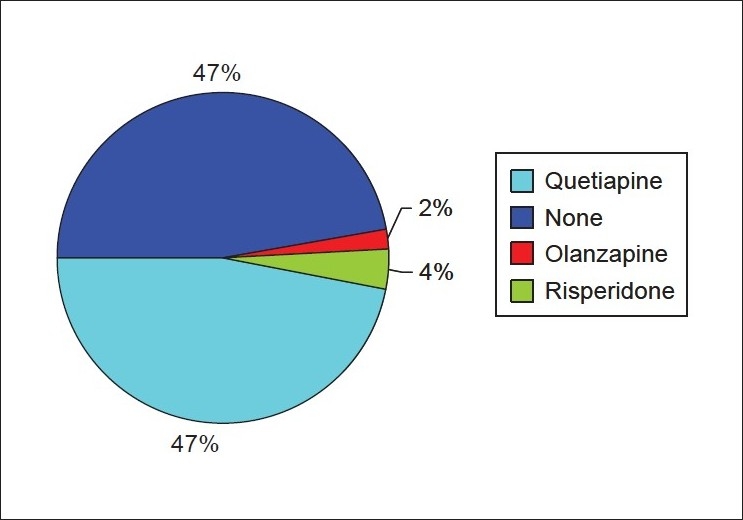
Pie diagram depicting the antipsychotics used in patients with dementia

The commonest antipsychotic used was Quetiapine, which was used in 24 patients (47%). It was used in a dose range of 25 to 300 mg/day. The duration of follow-up was up to 11 months (range: No follow-up to 11 months; average six weeks). A similar number of patients did not receive any antipsychotics. A minority of the patients received the other antipsychotics (ie., one patient received Olanzapine, two received Risperidone). The findings are depicted in Figure 2. None of the patients had adverse side effects with all three atypical antipsychotics, although sedation was reported by three patients in the Quetiapine group (12.5%). Four patients were prescribed selective serotonin reuptake inhibitors (SSRIs) and two patients received benzodiazepines.

## DISCUSSION

Dementias are common among elderly patients, but they are poorly recognized and treated in developing countries, including India.[10] To the best of our knowledge, there is no published and accessible research work to date, from India, looking into the prescription pattern in patients with dementia. This study shows that donepezil is the most commonly used antidementia agent, followed by memantine. The single daily dosing, lesser gastrointestinal side-effect profile, and cost of donepezil may have influenced the choice of prescription as compared to other choline-esterase inhibitors and memantine. The clinician's experience with the drug may also have influenced the choice of the antidementia drug. It is important to mention that the cost of antidementia drugs is entirely borne by the patients' family. This could be one of the probable reasons for not starting antidementia drugs for some patients.

In terms of comparative effectiveness, there is very little to choose from among the three choline-esterase inhibitors.[11] A systematic review found that the cost savings associated with reducing the mean time spent in full-time care did not offset the cost of treatment with choline-esterase inhibitors sufficiently, to bring the estimated cost-effectiveness to levels generally considered acceptable.[12] The same review remained inconclusive about the cost effectiveness of memantine. From the perspective of a developing country, a Taiwanese cost-effectiveness analysis of donepezil found it to be a cost-saving strategy for mild-to-moderate AD patients.[13]

The study found that quetiapine was the most common antipsychotic used for behavioral and psychotic symptoms. None of the patients were prescribed typical antipsychotics. The lesser propensity of quetiapine to cause extra pyramidal symptoms among the atypical antipsychotics may be the most plausible reason for prescribing it. The sedation that is commonly seen with quetiapine may be the other probable reason for the choice of the drug. The greater metabolic side effect profile of olanzapine and risperidone may also have deterred the use of these drugs in spite of them being cheaper. Antipsychotic drugs should be considered for BPSD only if there is a specific need, or if other treatments have failed; decision-making should be individualized and documented after a risk-benefit analysis. The results of the phase I outcomes from the Clinical Antipsychotic Trial of Intervention Effectiveness study for Alzheimer's disease (CATIE-AD) suggest that antipsychotics may be more effective for particular symptoms, such as anger, aggression, and paranoid ideas. They do not appear to improve the functioning, care needs, or quality of life.[14] The *Food and Drug Administration* (FDA), based on a meta-analysis of 17 double-blind randomized placebo-controlled trials among elderly people with dementia, commented that atypical antipsychotics were associated with a significantly (1.6-1.7 times) greater mortality risk when compared with placebos. However, there is a paucity of any other evidence-based treatment alternatives to antipsychotics for this population.[5] A watchful waiting strategy, entailing general medical management, nonspecific support, and delayed initiation of antipsychotic pharmacotherapy, has been advocated, in view of it being slightly less expensive and no less effective than immediate treatment with second generation antipsychotic medications.[15]

SSRIs were prescribed in a small minority of the patients. The patients who received SSRIs were those with fronto-temporal dementia. Only two patients received benzodiazepines, which were avoided probably due to risks such as falls and amnesia.

In summary, the study found donepezil and quetiapine to be the most commonly prescribed antidementia drug and antipsychotic, respectively. There are reasons to exercise caution against our study results as this was a small sample, and has to be taken with all the caveats of a retrospective chart-review design. Notwithstanding the above shortcomings, this investigation has indeed thrown up valuable results that will surely help fine tune the clinical prescription approach to dementia. The audit highlights the need to study the cost-effectiveness of antidementia and antipsychotic drugs for patients with dementia, in developing countries.
